# Bio-Inspired and Protein-Based Elastomeric Materials

**DOI:** 10.1021/polymscitech.5c00054

**Published:** 2025-06-09

**Authors:** Xiaodong Han, Juanjuan Su, Jingjing Li, Hongjie Zhang, Kai Liu

**Affiliations:** † Center of Materials Science and Optoelectronics Engineering, College of Materials Science and Optoelectronic Technology, 74519 University of Chinese Academy of Sciences, Beijing 100049, China; ‡ Engineering Research Center of Advanced Rare Earth Materials, Ministry of Education, Department of Chemistry, 12442 Tsinghua University, Beijing 100084, China; § 58277 Institute of Applied Chemistry, Changchun, Chinese Academy of Sciences, Key Laboratory of Rare Earth Resources Utilization, Changchun 130022, China; ∥ Xiangfu Laboratory, Building 5, No. 828 Zhongxing Road, Xitang Town, Jiashan, Jiaxing, Zhejiang, 314102, China

**Keywords:** Elastomeric material, Structural protein, Mechanical property, Artificially engineered protein, Biomedical application

## Abstract

Natural structural-protein-based elastomeric materials have garnered significant attention as potential alternatives to synthetic polymers, due to their remarkable biodegradability, biocompatibility, and low immunogenicity as well as their exceptional mechanical performance with high resilience, energy storage capacity, and fatigue resistance. However, the biosynthesis of artificially engineered elastomeric proteins and fabrication of elastomeric materials with on-demand properties remain great challenges, particularly in the context of designing and governing the hierarchical structure of protein assemblies. To understand the essence of mechanical properties and maximize the potential of protein-based elastomeric materials, this review systematically explores the sources and molecular mechanisms for elastic performance of a diverse range of natural elastomeric proteins. Subsequently, we discuss the design, biosynthesis, and rational assembly strategies of artificially engineered elastomeric proteins with specific secondary structures. Finally, we address the current challenges and provide perspectives on the development of next-generation protein-based elastomeric materials.

## Graphical abstract

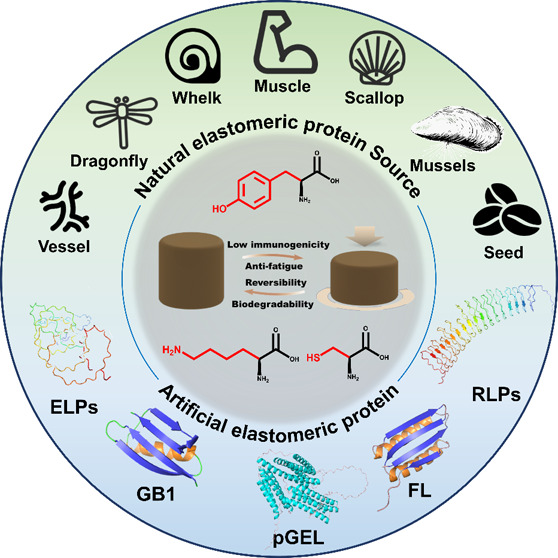

## 1 Introduction

Elastomeric materials, characterized by exceptional elasticity and the ability to recover rapidly from deformation, are indispensable for mechanically demanding applications. Traditionally, the development of elastomeric materials is greatly limited to chemically synthesized polymers, including polyurethane, poly­(ester ether), and styrene–butadiene, *etc*. These products are integral to many daily consumables, which has raised environmental concerns owing to the petroleum-based origins and limited recyclability.^1,2^ For instance, the ecological safety issues related to the deposal of nondegradable polymers and their accumulation in soil and marine environments seriously discourage the use of synthetic polymer elastomers. Additionally, some chemically synthesized polymers suffer from rigidity caused by *in vivo* hardening, poor biocompatibility, or biotoxicity, limiting their applications in the biomedical sector.^3^ To address these issues, biopolymers, particularly elastomeric proteins, have gained attention for their potential to provide sustainable, biocompatible alternatives with exceptional mechanical properties.

Elastomeric proteins are widely distributed across various animal tissues, and their precise structure and properties enable them to perform specific biological functions, including shock adsorption, and locomotion etc.^4^ These proteins exhibit unique rubber-like elasticity. They can maintain structural integrity even under large deformations, effectively storing elastic energy and rapidly reverting to their original conformation when unloaded. Integrating RNA-seq with proteomics facilitates the molecular characterization of natural elastomeric proteins. More importantly, the advance of DNA assembly technology and synthetic biology enables precise biosynthesis of engineered modular proteins,^5^ which contain recombinant functional domains derived from natural counterpart and endow tunable functions and mechanical properties to the ultimate protein-based elastomeric materials.^6–8^ Modular protein engineering offers an excellent toolbox for programming the protein sequence, physiochemical properties, secondary structures, and hence the development of elastomeric materials with desired properties and functions.^9–12^ Moreover, artificial intelligence and machine learning have recently been employed in the *de novo* design of structural proteins to guide the selection and modification of protein building blocks, and to mimic and even surpass the properties of natural protein elastomers.^13^ Besides, the combination of covalent and non-covalent cross-linking holds promise for creating elastomeric materials, which can seamlessly combine mutually incompatible mechanical properties including stiffness, toughness and recovery capability.^14–16^ However, the uneven cross-linking has remained an unsolved challenge, which might lead to batch-to-batch variations during material fabrication. Collectively, it remains challenging to manufacture protein elastomeric materials with predictive structural hierarchy and on-demand properties.

In this context, this review provides a comprehensive summarization of the sequence, structure, and mechanical properties of natural elastomeric proteins, as well as the development of artificial elastomeric proteins and materials for biomedical applications (Figure 1). We begin by exploring the relationship between the molecular structure and mechanical properties of elastomeric proteins from diverse natural sources, which provides a foundation for biomimetic design. Moreover, the biosynthesis and assembly of artificial elastomeric proteins are discussed, with an emphasis on specific secondary structures and cross-linking strategies. Finally, we conclude with forward-looking perspectives, highlighting key challenges and proposing promising research directions.

**Figure 1:**
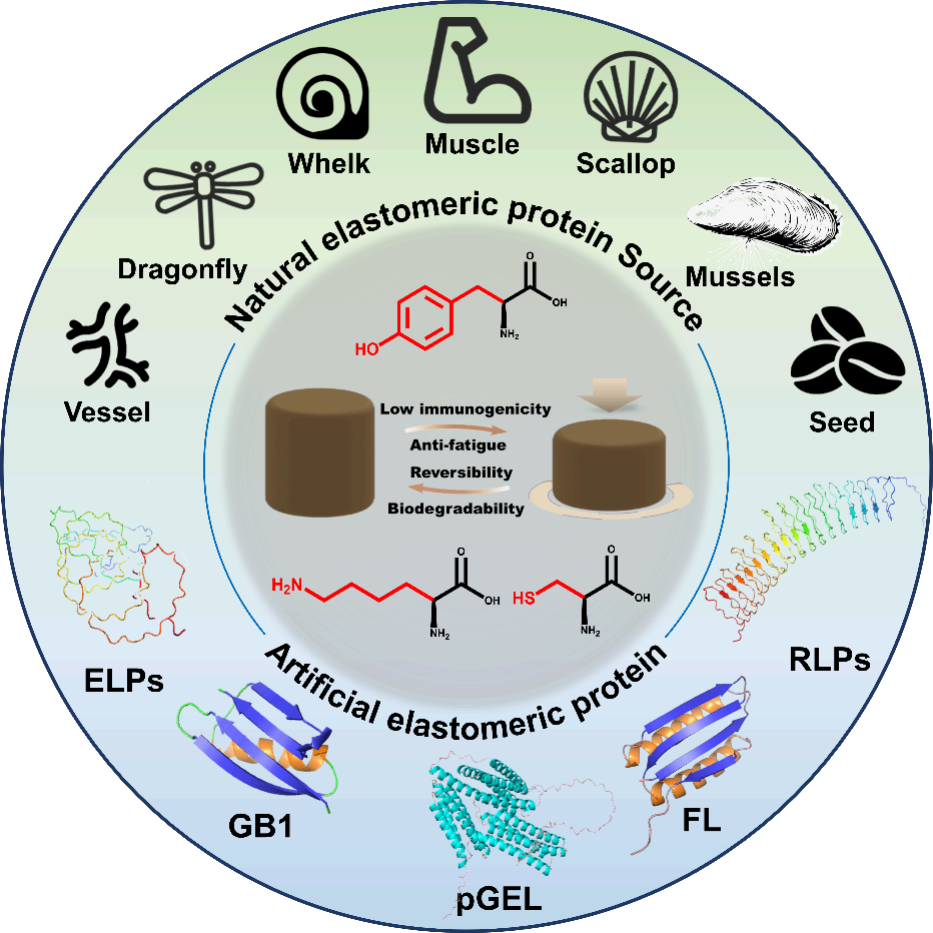
Schematic overview of bio-inspired and protein-based elastomeric materials, including natural elastomeric protein sources, artificial elastomeric protein structures, cross-linking methods, and performance characteristics.

## 2 Natural Elastomeric Proteins

Natural elastomeric protein materials consist primarily of structural proteins such as elastin, resilin, and titin, celebrated for their exceptional elasticity and ability to undergo reversible stretching. These elastomeric proteins play critical roles in various biological tissues, imparting essential elasticity to structures like blood vessels, skin, and muscles. Their physiological functions include enabling tissues to stretch and recoil, absorbing mechanical stress, and supporting flexibility and dynamic movement. The elasticity of these proteins stems from their unique structural features, which include repetitive amino acid sequences, hydrophilic and hydrophobic domains, and cross-linking mechanisms. These features facilitate reversible deformation, energy dissipation, and resilience against mechanical stress. Cooperative interactions such as disulfide bonds, hydrogen bonds, and other molecular interactions further enhance their mechanical properties, underscoring the pivotal role of their structure in determining functionality. Natural protein elastomeric materials are indispensable for a wide range of physiological processes due to their specialized structure and function.

### 2.1 Elastin

Elastin is a cross-linked protein located in the extracellular matrix,^17^ providing essential structural support and elastic recoil for the sustained stretching and recovery of soft, load-bearing tissues (Figure 2A-i). These tissues, such as blood vessels and skin, rely on elastin as a primary component. Elastic fibers, which elastin constitutes, typically range from 0.2 to 1.5 μm in diameter and often branch to form a loose connective tissue network. In denser elastic tissues such as the aorta, these fibers merge into flat sheets or elastic lamellae, further enhancing their mechanical properties.

**Figure 2:**
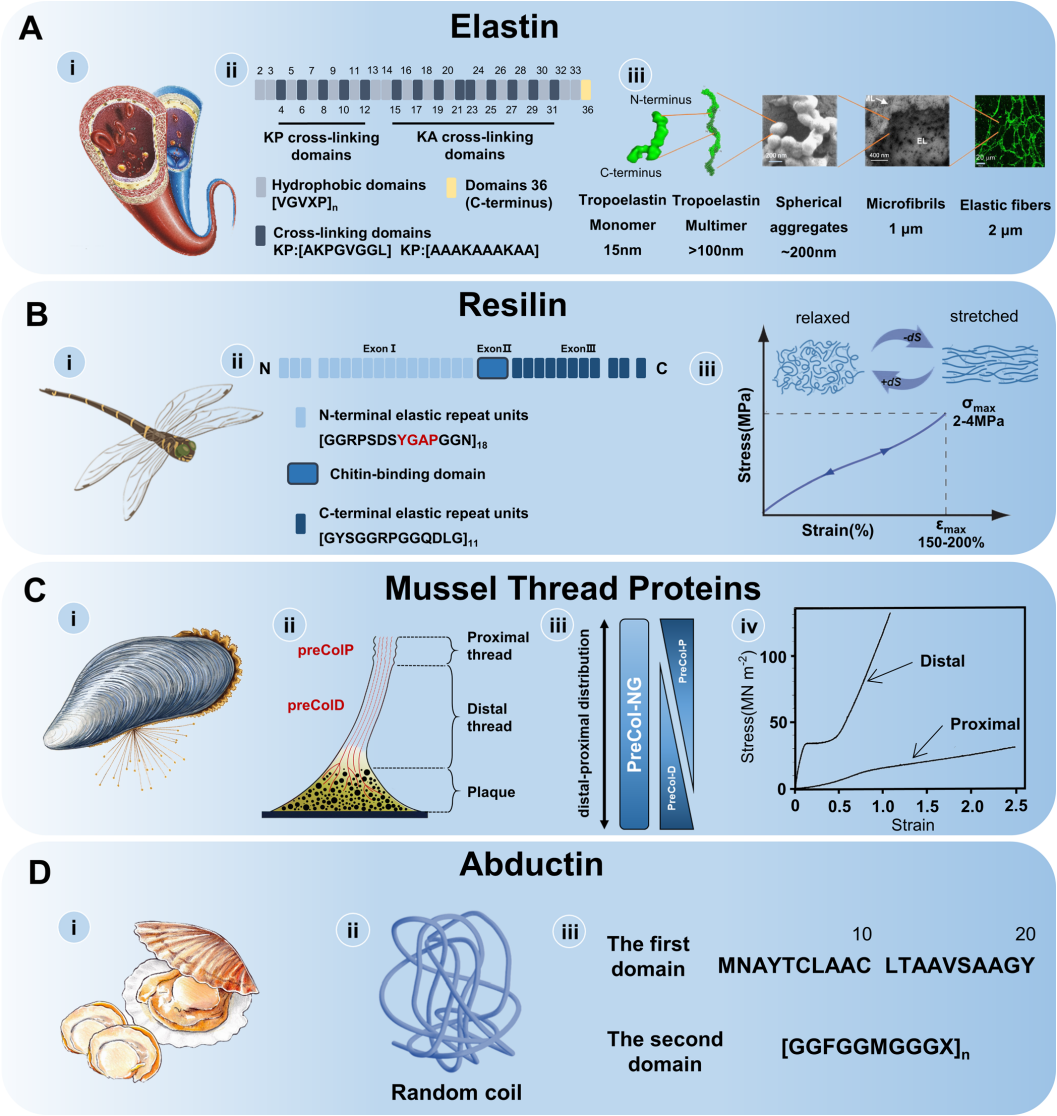
Proteins with entropy-driven elasticity. (A) (i) Human elastin provides elastic recoil for load-bearing tissues. (ii) The primary structure of tropoelastin. (iii) Formation of functional elastic fibers. Reproduced with permission from ref 20. Copyright 2021 Ozsvar, J. et al. Licensed under CC BY. (B) (i) In flying insects, the hinge of wings is made of the resilin protein. (ii) The primary structure of resilin. (iii) The typical stress–strain curve of resilin. Adapted with permission from ref 31. Copyright 2013 The Royal Society of Chemistry. Adapted with permission from ref 33. Copyright 2023 Wiley-VCH GmbH. (C) (i) Mussel byssal threads. (ii) The proximal region and distal region of mussel byssal fibers. Reproduced with permission from ref 34. Copyright 2024 Yulan Lyu et al. Licensed under CC BY 3.0. (iii) The core of the thread consists of three primary proteins—PreCol-P, PreCol-NG, and PreCol-D. (iv) The typical stress–strain curve of *M. californianus* mussel. Reproduced with permission from ref 35. Copyright 1996 The Company of Biologists Limited. (D) (i) Abductin is primarily found in the adductor muscles of scallops and other bivalves. (ii) Abductin has been found to possess a random coil secondary structure. (iii) Two domains of the abductin.

The precursor of elastin, tropoelastin, is a soluble 60–70 kDa protein that features two primary alternating domains (Figure 2A-ii): (1) hydrophilic cross-linking domains enriched with lysine and alanine, and (2) hydrophobic domains rich in valine, proline, and glycine, which are responsible for its elastic properties. These hydrophobic domains are often characterized by repetitive sequences such as VPGVG or VGGVG.^18^ Tropoelastin is secreted into the extracellular matrix, where it undergoes a highly organized assembly process involving liquid–liquid phase separation, aggregation, and enzymatic cross-linking.^19^ During this process, tropoelastin first aggregates into spherical droplets, which grow through coalescence and simultaneously undergo covalent cross-linking, facilitated by specific enzymes. Once mature, the elastin droplets are deposited onto a microfibril scaffold, where further aggregation and cross-linking lead to the formation of fully functional elastic fibers (Figure 2A-iii).^20^ This organized assembly of tropoelastin into functional elastic fibers is crucial for the mechanical properties of elastin, enabling tissues to maintain their elasticity and resilience under mechanical stress.

### 2.2 Resilin

Resilin is an elastomeric protein known for its remarkable elasticity,^21^ primarily found in various arthropods (Figure 2B-i), including locusts, dragonflies, mosquitoes, fruit flies, fleas, and others. It is present in structures such as joints, tendons, legs, mouthparts, and mechanical receptors.^22,23^ The main functions of resilin are to store elastic energy, reduce stress concentration, mitigate material fatigue, and confer flexibility to the organism.^24–26^ The full-length sequence of resilin was first reported in *Drosophila melanogaster* (Figure 2B-ii).^27^ The resilin sequence consists of three major regions: (1) The exon I,^28^ rich in glycine and proline, forms the elasticity-conferring domain. This region consists of 323 amino acids, including 18 pentadecapeptide repeats (GGRP­SDSY­GAPG­GGN or variants). (2) The exon II^27^ contains an extended RR-2 type Rebers & Riddiford consensus sequence (YDND­EPAK­YE FNYQ­VED­APS GLSF­GHS­EMR DGDF­TTG­QYN VLLP­DGR­KQI VEYE­ADQ­QGY RPQI­RYE­GDA NGG­SGP­SGP), critical for chitin binding. (3) The exon III^29^ consists of 235 amino acids, featuring 11 tridecapeptide repeats (GYSG­GRP­GGQ­DLG or variants). These exons together contribute to resilin’s unique mechanical properties. The elastic behavior of resilin can be described using the concept of entropic elasticity.^30^ In its highly disordered state, the resilin molecule exhibits high entropy, whereas elongation of the random chain through mechanical loading reduces its entropy. Consequently, the protein exhibits spontaneous recoil toward its initial, higher-entropy state. This is a key feature of resilin’s ability to undergo large deformations while maintaining a low modulus. The low hysteresis and high resilience of resilin, as demonstrated in the typical stress–strain curve (Figure 2B-iii), highlight its efficient energy storage and release during deformation.^31^ Notably, some variants of resilin, such as those found in fleas (Cf-resB), lack the chitin-binding domain in Exon II. In addition to these structural features, resilin contains a high tyrosine content, which facilitates the formation of dityrosine and trityrosine cross-links. Although covalent cross-linking typically restricts polymer chain mobility, resilin exhibits a unique architecture where sparsely distributed and flexible cross-links (e.g., dityrosine bonds) form a loosely connected network. This design stabilizes the overall structure while minimally impeding chain flexibility.^32^ Consequently, the intrinsically disordered chains retain their entropic elasticity, enabling both high extensibility and resistance to irreversible deformation. This molecular strategy explains the characteristic combination of low stiffness and high elasticity observed in natural resilin.

### 2.3 Mussel Thread Protein

Mussel byssal threads (Figure 2C-i) are sophisticated elastic fibers that exhibit gradient mechanical properties, enabling them to absorb and dissipate significant amounts of energy. These fibers are exceptionally tough, capable of enduring strains up to 200% and recovering from them, while dissipating up to 70% of the absorbed energy,^35^ which is highly desirable from a biomimicry standpoint. This performance is due to the gradient mechanical properties engineered into the structure of the threads, which consist of distinct distal and proximal segments^36^ (Figure 2C-ii). The thread core consists of a family of modular collagenous proteins known as preCols. The central preCol domain contains a characteristic (Gly-X-Y)_n_ repeat sequence, which is typical of fibrillar collagen, where X and Y are frequently proline or hydroxyproline. Surrounding the collagen domain are the flanking regions, which vary among the three identified preCol variants. PreCol-P, found at the proximal end, has a highly conserved pentapeptide motif GPGGG, which is similar to the hydrophobic sequence of elastin. PreCol-D, located at the distal end, features dragline silk-like flanking domains, with polyalanine stretches and glycine-rich spacers. The PreCol-D domain serves as a reversible hidden-length reservoir, significantly contributing to the protein extensibility.^37^ PreCol-NG, which is uniformly distributed, has a glycine-rich sequence resembling the extensible domains found in flagelliform silk. Both the N- and C-termini of all preCols are characterized by histidine-rich domains (HRDs), which are composed of approximately 20 mol % histidine residues interspersed throughout.

The material properties of *M. californianus* mussel are indicated as the stress–strain curves in Figure 2C-iii.^38^ The proximal region is highly extensible, with a strain of approximately 200%, indicating rubber-like behavior. However, its stiffness and strength are about an order of magnitude higher than elastin and arthropod resilin. The distal region exhibits slightly lower extensibility, around 100%, but its stiffness and strength are comparable to collagen. Due to its combination of high strength and extensibility, the toughness of both the proximal and distal byssal threads is roughly an order of magnitude greater than that of arthropod resilin or elastin, and approximately six times greater than tendon collagen. The toughness of byssal fibers is on par with that of Kevlar and carbon fibers, and this exceptional toughness is undoubtedly critical for the survival of mussels in the marine intertidal zone.

### 2.4 Abductin

Native abductin was identified in the abductor ligament firstly, a structure located in the internal hinge of bivalves such as scallops and clams (Figure 2D-i). This protein exhibits rubber-like properties and behaves similarly to a coiled spring (Figure 2D-ii).^39^ Upon relaxation of the adductor muscles, the abductin facilitates the opening of the tightly closed shell. This mechanism is essential for the locomotion of scallops, as it enables the shell to open at a frequency of 4 Hz, allowing the scallop to swim several meters in a single movement, thereby evading slow-moving predators. In terms of mechanical properties, native abductin exhibits a tensile modulus of 1.25 MPa, which is higher than that of elastin^40^ (0.3–0.6 MPa) but comparable to that of resilin (0.6–2 MPa). Furthermore, its compression modulus is 4 MPa, exceeding that of resilin^41–43^ (0.6–0.7 MPa). These mechanical characteristics suggest that native abductin is well-suited to withstand both tensile and compressive forces, which is critical for its role in the dynamic function of the abductor ligament.

The primary structure of abductin was first deduced by Cao et al.^44^ (based on the cDNA sequence of the bay scallop, *Argopecten irradians*) and is organized into two distinct domains (Figure 2D-iii). The first domain, an alanine-rich amino-terminal region (residues 1–20), contains two conserved cysteine residues (positions 6 and 10), which may be involved in the formation of intermolecular or intramolecular disulfide bonds, contributing to the protein’s structural stability. It also includes two of the three conserved tyrosine residues (positions 4 and 20), likely involved in cross-linking through the formation of 3,3′-methylenebis­(phenylene)­benzene.^45^ The second domain is characterized by a glycine/methionine-rich sequence, where methionines are distributed throughout the domain and are included in the consensus sequence GGFG­GMG­GGX. These structural features may influence the mechanical properties of abductin, particularly its role in energy storage and dissipation. Due to its high glycine content, abductin is likely to maintain an overall random coil structure (Figure 2D-ii). Unlike other rubber-like proteins that are rich in proline and prone to forming secondary structures such as β-turns, abductin contains a very low proline content and is unlikely to adopt such conformations. Therefore, its elasticity is primarily derived from a network of randomly coiled protein chains, consistent with the principles of rubber elasticity theory.^46^ Classical rubber elasticity theory posits that the mechanical behavior of elastomeric networks is governed by entropy-driven forces rather than internal energy changes. In equilibrium, polymer chains adopt random coil conformations. Mechanical deformation induces chain alignment and extension, reducing configurational entropy. Upon force removal, the system spontaneously recovers its high-entropy state, producing a restoring elastic force.^47^ These features can be accurately modeled using statistical thermodynamic approaches such as the Neo-Hookean, Mooney–Rivlin, or Gent models, which describe the elastic response of cross-linked polymer networks as a function of chain density, temperature, and deformation.^48^ Likewise, abductin elasticity can be explained within this framework, reflecting the fundamental physical principles governing the mechanical behavior of intrinsically disordered protein networks.

### 2.5 Egg Capsules Protein

The egg capsules of certain marine gastropods (Figure 3A-i) exhibit extraordinary long-range reversible elasticity, driven by unique molecular deformation mechanisms (Figure 3A-ii). These capsules provide essential protection for developing embryos against various environmental stressors, including mechanical impacts, chemical threats, and hydration-dehydration cycles during tidal fluctuations. The mechanical properties of the egg capsules, first characterized by Rapoport and Shadwick,^49^ display distinct stress–strain behaviors (Figure 3A-iii). The mechanical testing revealed an initial linear elastic region with a high modulus of 50–100 MPa, followed by a pseudo-yield state at 4–5% strain, a plateau phase with moderate stress increase, and a strain-hardening region above 70% strain leading to a stress peak. Upon unloading, significant stress–strain hysteresis was observed, indicating energy dissipation.^50^ This phenomenon is critical because the material loses a considerable amount of energy during deformation, enhancing its shock-absorbing capability and protective performance. This property underscores the material’s cushioning and shock-absorbing functions, which are critical for the survival of embryos during prolonged development.^51^

**Figure 3:**
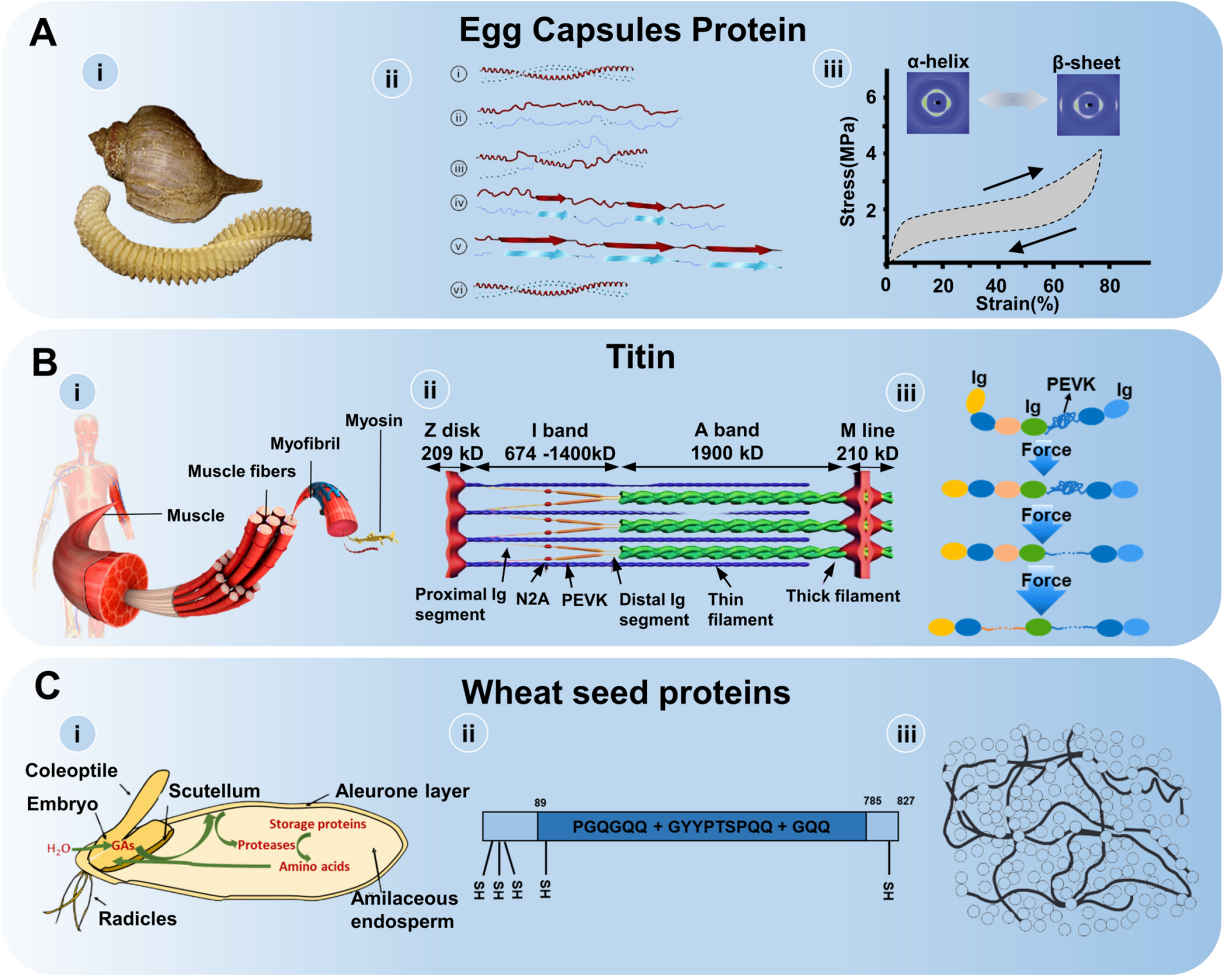
Elastomeric protein involving structural transitions and energy changes. (A) (i) Egg capsules of certain marine gastropods. Reproduced with permission from ref 50. Copyright 2015 The Royal Society of Chemistry. (ii) The transformation of α- helix and β-sheet. Reproduced with permission from ref 54. Copyright 2009 Springer Nature Limited. (iii) The distinct stress–strain behaviors. Adapted with permission from ref 50. Copyright 2015 The Royal Society of Chemistry. (B) (i) Muscle comprises of muscle fibers, myofibrils, and the smallest units, known as sarcomeres. Reproduced with permission from ref 55. Copyright 2021 Nobuto Nakanishi et al. Licensed under CC BY. (ii) In the sarcomere (lower left), titin connects actin-containing thin filaments and myosin-containing thick filaments. Reproduced with permission from ref 56. Copyright 2012 The Royal Society. (iii) The mechanical stabilities of Ig domains in titin revealed by force–extension experiments. (C) (i) Wheat seed proteins. Reproduced with permission from ref 57. Copyright 2019 Mercedes Diaz-Mendoza et al. Licensed under CC BY 4.0. (ii) The three domains of the wheat seed protein. (iii) The “ring” and “chain” regions of the gluten structure. Reproduced with permission from ref 58. Copyright 1999 Academic Press.

At the molecular level, the egg capsule is composed of a protein elastomer with an α-helical structural motif, as revealed by X-ray diffraction studies. Under strain, the α-helical coils progressively unwind and transition into extended β-sheets aligned with the loading direction, accommodating strains of up to 150%. Upon unloading, the β-sheets revert to their original α-helical conformation, enabling full structural recovery (Figure 3A-ii). This reversible transformation is driven primarily by changes in internal energy, rather than entropy, and represents a unique elastic mechanism. The elasticity of egg capsule proteins is governed by an activation enthalpy-mediated conformational change mechanism (Figure 3A-iii), rather than entropic chain recoil.^52^ The α-to-β transition involves breaking and reforming of hydrogen bonds and backbone rearrangements, requiring a threshold level of mechanical work to initiate. The energy stored during stretching is thus partially retained in the higher-energy β-sheet conformation and released upon recovery. This internal energy–dominated mechanism reflects a form of phase transition elasticity, where the mechanical behavior is tightly coupled to molecular-level energetics rather than chain configurational entropy.

The egg capsule proteins (ECPs) are built around a central rod domain comprising two or three coiled-coil helical regions. These regions contain consecutive heptad repeat sequences *abcdefg*, where hydrophobic residues at positions “a” and “d” stabilize the helical core.^53^ The helical domains are interspersed with flexible linker sequences, while the N- and C-termini form the head and tail domains. The combination of reversible elasticity, energy dissipation, and shock absorption observed in egg capsules underscores their biological significance and offers a valuable model for the design of bio-inspired materials. These findings hold potential for applications in impact-resistant coatings, tissue scaffolds, and other biomimetic engineering fields.

### 2.6 Titin

Titin, the largest protein known in nature, was first reported by R. Natori and Y. Nonomura.^59^ This giant protein spans half of the sarcomere, the fundamental contractile unit of striated muscle (Figure 3B-i), and plays a pivotal role in muscle elasticity and structural integrity.^60^ The elastic region of titin is located within the I-band of the sarcomere, a segment whose length varies across muscle types. Titin is composed predominantly (∼90%) of globular domains resembling immunoglobulin (Ig) or fibronectin type III (FN_3_)-like folds, with the remaining mass consisting of unique sequence insertions. Three major isoforms of titin have been identified, differing in their I-band compositions:^61^ N_2_B (∼3.0 MDa) and N_2_BA (3.2–3.7 MDa) in cardiac muscle and N_2_A (∼3.3–3.7 MDa) in skeletal muscle (Figure 3B-ii). Despite these variations, all isoforms share a conserved structural framework, including tandemly arranged Ig domains in the proximal (I_1_–I_15_) and distal (I_84_–I_105_) regions, as well as a PEVK domain, named for its high content of proline (P), glutamate (E), valine (V), and lysine (K) residues.

The mechanical properties of titin arise from the unique conformations and behaviors of its structural domains. Ig domains, comprising ∼90–100 amino acids arranged in a β-sheet structure, provide stability and modular elasticity. In contrast, the PEVK domain adopts a flexible, random coil conformation, which elongates under mechanical strain. Titin exhibits a staged response to external force,^62^ as shown in Figure 3B-iii. At lower forces (on the order of a few tens of picoNewtons), the Ig domains straighten, a process referred to as “tertiary elasticity”. With increasing force, the disordered PEVK segment extends, contributing further to titin’s elasticity. Under high strain, individual Ig domains begin to unfold sequentially, a phenomenon known as “secondary elasticity”. This hierarchical mechanism enables titin to withstand and dissipate significant mechanical stress while maintaining its functional integrity during repeated cycles of muscle contraction and relaxation. This remarkable adaptability underscores titin’s critical role in muscle function, providing both elasticity and structural support in response to varying mechanical demands.

### 2.7 Wheat Seed Protein

Wheat seed proteins (Figure 3C-i) account for approximately 50% of the total protein content in dry grains. These proteins are unique among cereal proteins due to their ability to form a continuous network when flour is hydrated and kneaded, imparting elasticity to the dough.^63^ This property is essential for the preparation of bread and other baked products. Although gluten consists of more than 50 different proteins, a subset of 3–6 high-molecular-weight proteins is primarily responsible for its elastic behavior. Each subunit comprises three domains:^64^ short non-repetitive N-terminal (88-104 residues) and C-terminal (∼42 residues) regions that flank an extended repetitive central domain (440-680 residues). The central domain contains three repeating sequence motifs (Figure 3C-ii): hexapeptides (PGQGQQ), non-peptides (GYYPTSP/LQQ), and tripeptides (GQQ), which are enriched in proline, glycine, and hydrophilic residues, especially glutamine.

The elasticity of gluten relies on the cooperative action of disulfide bonds and non-covalent hydrogen bonds. Disulfide bonds provide an elastic framework for the gluten chains, while hydrogen bonds, particularly those involving glutamine residues, are crucial for stabilizing the structure and contributing to its elasticity. Studies indicate that, under low hydration conditions, gluten proteins predominantly adopt β-sheet structures, with interchain hydrogen bonds stabilizing close interactions. As hydration increases, the β-sheet content decreases, some hydrogen bonds break, and new bonds form between glutamine residues and water molecules. This shift results in a dynamic balance between the “ring” and “chain” regions of the gluten structure (Figure 3C-iii), facilitating elastic energy storage during stretching.^58^ This process supports the dough’s extensibility and resistance to deformation. Furthermore, esterification of glutamine residues weakens hydrogen bonding, leading to reduced ductility, while stirring in deuterium oxide strengthens hydrogen bonds and increases extension resistance. These findings underscore the critical role of hydrogen bonds in the elastic properties of gluten.

## 3 Bio-Inspired Artificial Elastomeric Proteins and Assembled Materials

Natural elastomeric proteins play a crucial role in various physiological functions. With the rapid advancement of synthetic biology and genetic engineering, researchers have increasingly drawn inspiration from these natural proteins to design and synthesize artificial elastomeric proteins, aiming to create materials with comparable or even superior properties. Artificial elastomeric proteins not only inherit the exceptional mechanical properties and biological advantages of natural proteins but also offer significant design flexibility. This flexibility allows for the adjustment of mechanical properties, biodegradability, and cellular interactions to meet specific application requirements. The diverse amino acid composition in proteins provides multiple sites for the preparation of elastomeric materials. Lysine residues enable rapid cross-linking via Mannich-type condensation to form elastomeric materials, while tyrosine cross-linking enhances rigidity and toughness through the formation of dityrosine bridges. Cysteine residues, on the other hand, promote the formation of disulfide bonds under oxidative conditions, improving ductility and toughness.^65^ Furthermore, the secondary structure of the protein—such as random coils, α-helices, and β-sheets—plays a critical role in determining the material’s mechanical properties, including elasticity, tensile strength, and ductility (Table 1).

The resilience of elastomeric protein materials mainly arises from the synergistic effects of multiple mechanisms, such as entropic elasticity, reversible secondary structure transformation (e.g., α-helices to β-sheets), and hydration of protein chains. In contrast, the resilience of chemically synthesized elastomers such as polyurethane and rubber depends on the entropic recovery of polymer segments and the network constraints provided by physical cross-linking or microphase separation structures.

**Table 1:** Comparison of Mechanical Properties of Elastomeric Materials Derived from Engineered Proteins and Synthetic Polymer

Type	Samples	Secondary structure	Resilience (%)	Modulus (KPa)	Extensibility (%)	Cross-linking methods	Ref.
Engineered resilin	**Pro-resilin**	Random coil	97 (at 225% strain)	25	313	Ru(II)-mediated photo-initiated cross-linking	^66^
	**R**_**4**_ **S**_**8**_	Random coil	>95 (at 60% strain)	-	158.2 ± 21.9	Physical cross-linking and photo-initiated cross-linking	^67^
	**RLP**_**12**_	Random coil	95–99 (at ≤100% strain)	30.3 ± 8.1	192 ± 22	Mannich reaction	^68^
Engineered elastin	**KCTS-E**_**31**_ **-KCTS**	Random coil	64.87 ± 2.55 (at 70% strain)	2.21 ± 0.36	419 ± 25	UV mediated photo-initiated cross-linking	^69^
	**MeTro**	Random coil	98 (at 50% strain)	39.7 ± 3.8	∼60	UV mediated photo-initiated cross-linking	^70^
	**V50CK1**	Random coil	94 (at 600% strain)	19	1530	Michael addition	^71^
Other engineered proteins	**(FL)**_**8**_	β-sheet	-	700 ± 110	107 ± 14	Chain entanglements and photo-initiated cross-linking	^72^
	**(G–R)**_**4**_	Mixed secondary structures	>90 (at <15% strain)	70	35	Ru(II)-mediated photo-initiated cross-linking	^73^
	**GRG**_**5**_ **RG**_**4**_ **R**	Mixed secondary structures	>95 (at <15% strain)	50	135	Ru(II)-mediated photo-initiated cross-linking	^73^
Synthetic polymers	**Polyurethane**	-	60–80	1000–100,000	300–800	Physical and covalent hybrid cross-linking	^74^
	**Rubber**	-	60–70 (at 100–130% strain)	14,000	300–400	Free radical polymerization	^75^

### 3.1 Elastomeric Materials from Proteins with Random Coil Structures

#### 3.1.1 Elastomeric Materials Based on ELPs

Elastin’s unique structure and behavior have led to the development of elastin-inspired molecules, with elastin-like polypeptides (ELPs) being the most extensively studied. ELPs are biopolymers made up of short repeating peptide motifs, primarily the pentapeptide VPGXG, where X represents a guest residue that can be any amino acid except proline.^10^ ELPs exhibit thermoresponsive behavior (Figure 4A), transitioning from an extended random coil conformation to a β-spiral structure as the temperature increases, resulting in phase separation and exposure of hydrophobic side chains.^76^ This behavior, characterized by a lower critical solution temperature (LCST), can be precisely tuned by modifying the guest residues and peptide chain length, which makes ELPs highly versatile for various applications such as drug delivery, surface modification, nanoparticle formation, and tissue engineering.

**Figure 4:**
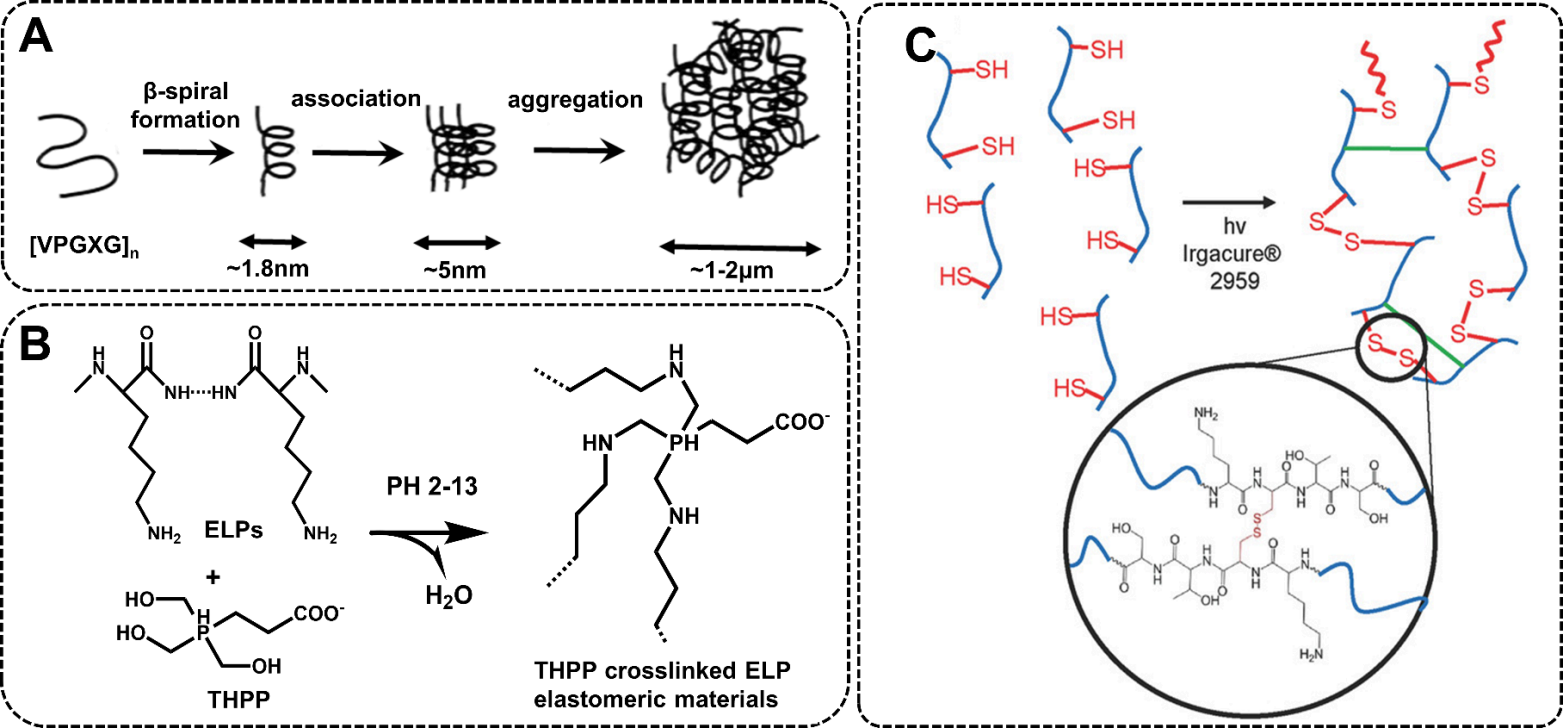
(A) The ELP aggregation mechanism. Reproduced from ref 76. Copyright 2014 Tomasz Kowalczyk et al. Licensed under CC BY. (B) Schematic of the inter- or intramolecular cross-linking mechanism between Lys residues of ELPs and THPP. Reproduced with permission from ref 77. Copyright 2007 American Chemical Society. (C) The photo-initiated cross-linking mechanism for cysteine-containing ELP (KCTS-E_31_-KCTS). Reproduced with permission from ref 78. Copyright 2015 Wiley-VCH.

The mechanical properties of ELPs can be further enhanced through cross-linking, particularly by modifying lysine residues. Lysine-based cross-linking is commonly achieved using bifunctional reagents, such as glutaraldehyde, hexamethyl isocyanate, and bis­(sulfosuccinimidyl) suberate. These cross-linked ELPs exhibit elastic properties with moduli similar to native elastin, ranging from 0.3 to 0.6 MPa, and elongations of 200-400%.^79,80^ The mechanical properties can be fine-tuned by adjusting factors such as the molecular weight of the ELP, the spacing and location of lysine residues, the ratio of cross-linker to primary amines, and the choice of cross-linker. However, their application is limited due to the potential toxicity of cross-linking agents. Furthermore, a Mannich-type condensation reaction using β-[tris­(hydroxylmethyl)­phosphino]­propionic acid (THPP) has been explored for cross-linking lysine-containing ELPs,^81^ offering a biocompatible and water-soluble method for creating injectable biomaterials (Figure 4B). Under physiological conditions, THPP-induced cross-linking results in elastomeric biomaterial with shear moduli comparable to those of certain connective tissues, such as the nucleus pulposus or menisci. The biomaterials exhibit non-cytotoxicity and good biocompatibility, making them promising candidates for use in tissue regeneration applications, particularly in load-bearing environments such as small-diameter vascular grafts. Ali et al.^78^ reported a photo-initiated cross-linking system that can overcome the limitations of chemical cross-linking, as shown in Figure 4C. They constructed a cysteine-containing ELP (KCTS-E_31_-KCTS) that can form elastic materials through photo-initiated cross-linking under UV light in the presence of the photoinitiator Irgacure®2959. Cysteine serves two functions: extending the protein chain and forming disulfide bonds as a cross-linker. This material exhibits high ductility (up to 420%) and adjustable tensile strength, making it suitable for engineering elastic tissues such as blood vessels, skin, lungs, or heart tissue.

To conclude, the versatile nature of ELPs, combined with tunable cross-linking strategies and their excellent biocompatibility, makes them highly promising materials for a wide range of biomedical applications, particularly in tissue engineering and regenerative medicine.

#### 3.1.2 Elastomeric Materials Based on RLPs

In 2001, Ardell and Andersen^82^ identified the gene sequence of resilin (CG15920) in the fruit fly *Drosophila melanogaster*, paving the way for the engineering of resilin-like polypeptides (RLPs) with properties similar to natural resilin. In recent years, numerous RLPs have been developed through molecular cloning techniques (Table 2), garnering significant interest in the scientific community due to their unique characteristics, such as multi-responsiveness and potential for diverse applications.

**Table 2:** Origin and Sequence Characteristics of Different RLPs

Source	RLPs	Description	Ref.
Fruit fly (*D. melanogaster*)	Rec1-resilin	18 pentadecapeptide repeats (GGRPSDSYGAPGGGN)	^83^
	Dros16	16 repeats of a consensus sequence (GGRPSDSYGAPGGGN)	^84^
	RLP-X (X = 12, 24, 36, or 48)	12, 24, 36, or 48 repeats of a consensus sequence (GGRPSDSFGAPGGGN)	^85^,^86^
African malaria mosquito (*A. gambiae*)	An16	16 repeats of a consensus sequence (AQTPSSQYGAP)	^87^
	RZ10-X (X = RGD or RDG)	10 repeats of a consensus sequence (AQTPSSQFGAP)	^88^
Flea (*Ctenocephalides felis*)	Cf-resB	Isoform B transcript of full length resilin devoid of a chitin-binding domain	^89^
Buffalo fly (*Haematobia irritans exigua*)	Hi-resB	Isoform B transcript of full length resilin devoid of a chitin-binding domain	^89^

Natural resilin is a cross-linked biopolymer, with dityrosine and trityrosine cross-links formed between its tyrosine residues, contributing to its exceptional elasticity. These cross-links are crucial for the rubber-like elasticity characteristic of resilin. To replicate the properties of natural resilin, resilin-like polypeptides (RLPs) can be cross-linked through physical or chemical methods to produce elastomeric materials with similar properties. Two commonly used tyrosine-based cross-linking methods have been developed for producing elastomeric protein materials: one involves ruthenium compounds, while the other utilizes the horseradish peroxidase (HPO)-hydrogen peroxide (H_2_O_2_) system. Both methods rely on the formation of covalent dityrosine cross-links to enhance the mechanical properties of the resulting biomaterials.

The first method involves Ru­(II)-mediated photo-initiated cross-linking, which utilizes ruthenium complexes known for their excellent electrochemical and photochemical properties, in combination with ammonium persulfate. Upon visible light irradiation (450 nm), photoexcited Ru­(II) complexes transfer electrons to electron acceptors, leading to the oxidation of Ru­(II) to Ru­(III). The resulting Ru­(III) subsequently oxidizes tyrosine residues in the protein, generating unstable tyrosine radicals that couple to form dityrosine molecules, which are further stabilized by persulfate radicals (Figure 5A-i). Lyons et al.^87^ reported the Ru­(II)-mediated photo-initiated cross-linking of An16 and Dros16, resulting in cross-linked elastomeric materials with resilience of approximately 94% and 91% (Figure 5A-ii), respectively. The HPO–H_2_O_2_ system offers a biocatalytic alternative for cross-linking. HPO is a heme-containing enzyme that catalyzes the oxidative conjugation of phenol and aniline derivatives in the presence of H_2_O_2_, forming dityrosine bridges.^92^ Qin et al.^91^ employed this method to cross-link proteins encoded by exons 1 and 3 of the *Drosophila melanogaster* resilin gene, yielding rubber-like biomaterials, as shown in Figure 5B. Atomic force microscopy (AFM) analysis revealed that the protein from exon 1 exhibited 90% elasticity, while the protein from exon 3 demonstrated 63% elasticity.

**Figure 5:**
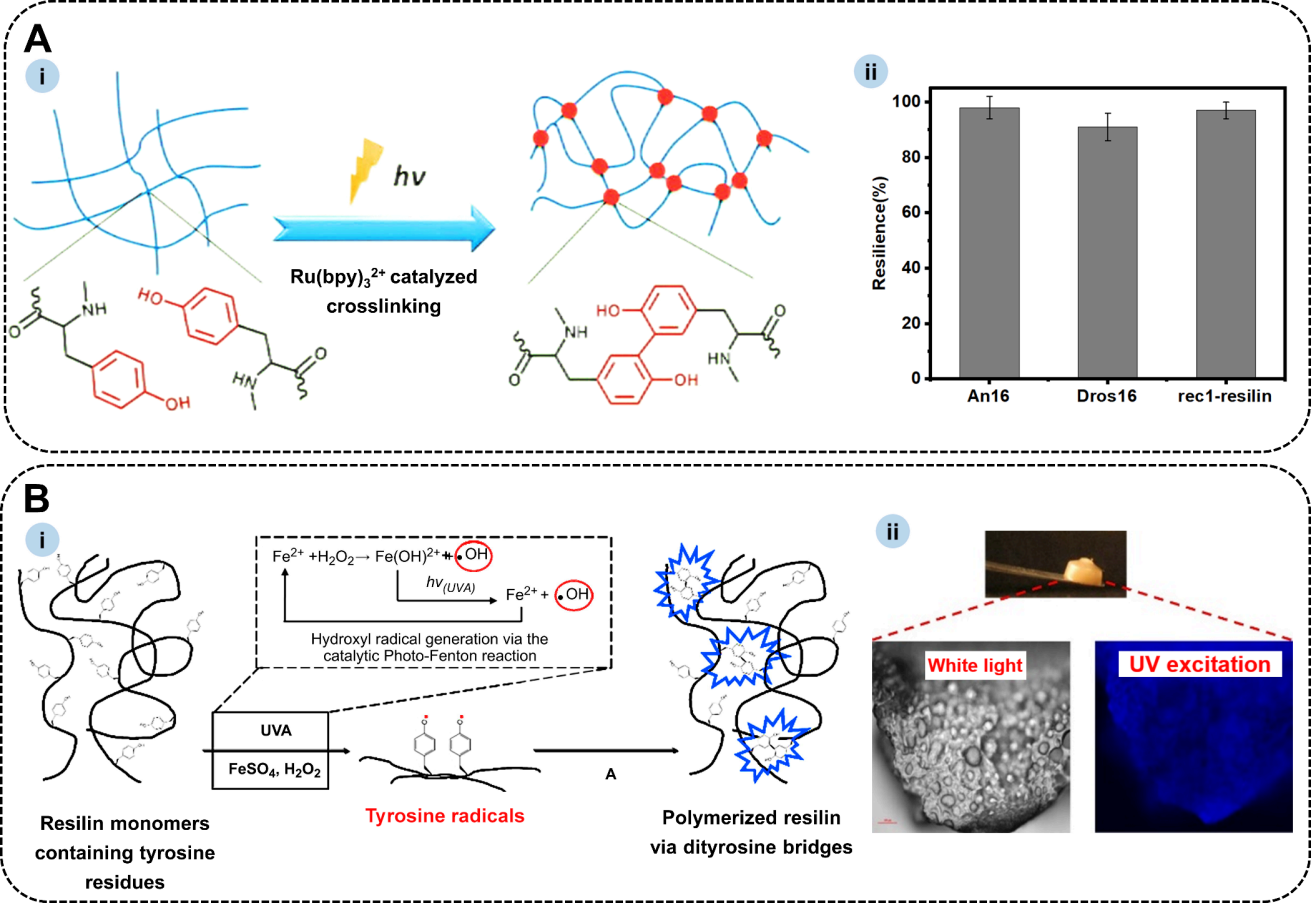
(A) (i) Schematic of Ru­(bpy)_3_^2+^-mediated photo-initiated cross-linking. Reproduced with permission from ref 90. Copyright 2013 American Chemical Society. (ii) The resilience of An16, Dros16, and rec1-resilin. (B) (i) Schematic diagram of tyrosine cross-linking via the HPO–H_2_O_2_ system. (ii) Rubber-like biomaterials formed by tyrosine cross-linking. Reproduced with permission from ref 91. Copyright 2011 Elsevier Ltd.

These cross-linked RLP-based elastomeric materials exhibit ideal rubber-like elasticity. The mechanical properties of the gels, such as enhanced elastic modulus and reduced creep strain, can be tuned by adjusting the cross-linking density. With physicochemical properties that mimic the extracellular matrix, RLP elastic materials support cell growth and hold great potential for applications in vascular/cardiovascular prosthetics and vocal cord tissue engineering.

### 3.2 Elastomeric Materials from Proteins with an α-Helix

Talin is a quintessential mechanoprotein that plays a crucial role in linking the actin cytoskeleton to integrin receptors in the extracellular matrix, serving as a mechanosensor. When stretched within a physiologically relevant range, talin maintains the average force exerted on the protein below 10 pN by undergoing unfolding and refolding events in its 13 tetrahelical or pentahelical rod domains.^93^ Notably, upon the removal of force, the refolding of the talin rod domain occurs with high fidelity over multiple force cycles, demonstrating that talin functions as a cellular shock absorber. To further explore its potential, we engineered a recombinant version of talin, called pGEL, which consists of the three rod domains (R1–R3) of talin.^94^ These domains were modified by mutating internal cysteine residues to serine and introducing cysteine residues at both ends of the protein, enabling it to form polymers as monomers. The resulting protein gel was prepared using a Michael addition reaction, which allows it to unfold and refold under mechanical force (Figure 6A). Due to the presence of an unfolding-refolding energy dissipation mechanism, the protein gel is capable of withstanding the impact of supersonic bullets, making it a promising shock-absorbing material for applications in the aerospace and automotive industries. Therefore, recombinant folded proteins can efficiently facilitate energy conversion through reversible conformational transitions, providing protection and recovery for deformed materials (Figure 6B).

**Figure 6:**
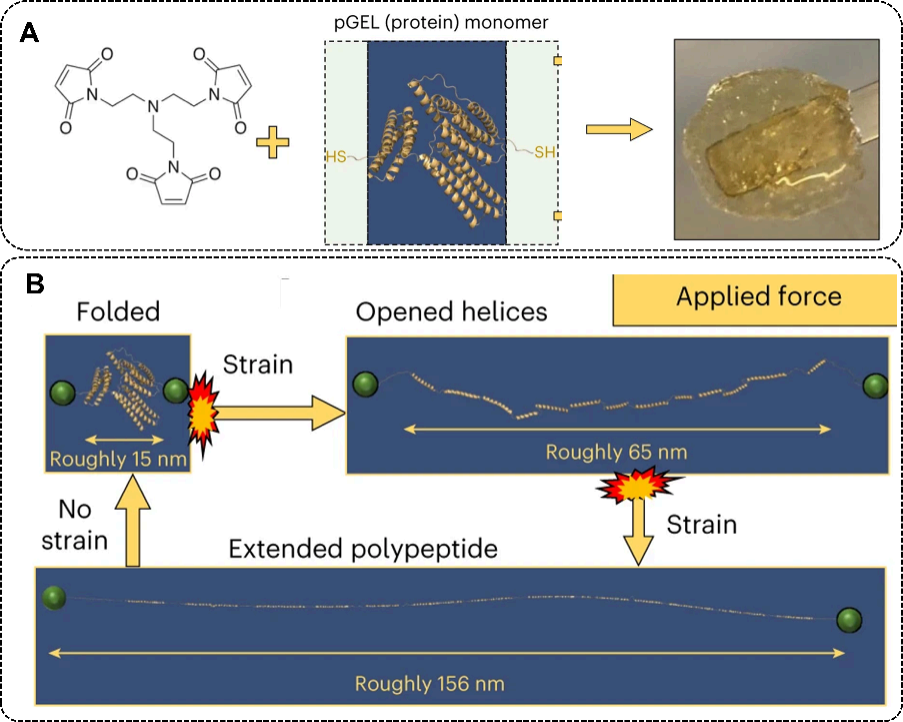
(A) Schematic diagram of the preparation of the talin-based biomaterial. (B) Schematic diagram of the shock absorption mechanism. Reproduced with permission from ref 94. Copyright 2023 Jack A. Doolan et al. Licensed under CC BY 4.0.

### 3.3 Elastomeric Materials from Proteins with β-Sheets

#### 3.3.1 Elastomeric Materials Based on GB1

Naturally occurring elastomeric proteins serve as molecular springs within their physiological environments, establishing elastic connections that provide mechanical strength, elasticity, and extensibility.^95^ These proteins also exhibit remarkable reliability in repeated stretching. Inspired by the extraordinary properties of natural elastomeric proteins, particularly titin, researchers have sought to explore non-mechanical proteins as part of efforts to expand the elastomeric protein toolbox. One such example is the streptococcal G protein, specifically the B1 immunoglobulin domain^96^ (GB1), a small α/β protein composed of 56 amino acids. The GB1 domain forms a compact globular structure, with a four-stranded β-sheet adjacent to a long α-helix, a structural arrangement that plays a crucial role in its mechanical stability. Li et al. demonstrated the high mechanical stability of a multimeric form of GB1, denoted as (GB1)_8_, which consists of eight tandem repeats of the GB1 domain. Single-molecule atomic force microscopy (AFM) stretching of the (GB1)_8_ protein yielded a characteristic sawtooth force-stretch curve. The average folding force of the GB1 domain was approximately 180 pN,^97^ a value comparable to the mechanical stability of the I27 domain of the natural elastomeric protein titin (Figure 7A-i). The β-sheets at both terminal ends of the GB1 domain are connected by hydrogen bonds, conferring resistance to mechanical unfolding. This structural feature is pivotal in ensuring the domain’s high mechanical stability. Artificial GB1 polyproteins exhibit distinct mechanical properties, including rapid and precise folding kinetics, resistance to mechanical fatigue, and the ability to refold against residual forces. These properties facilitate the efficient recovery of mechanical stability and significantly mitigate fatigue during extended stretch-relaxation cycles.

**Figure 7:**
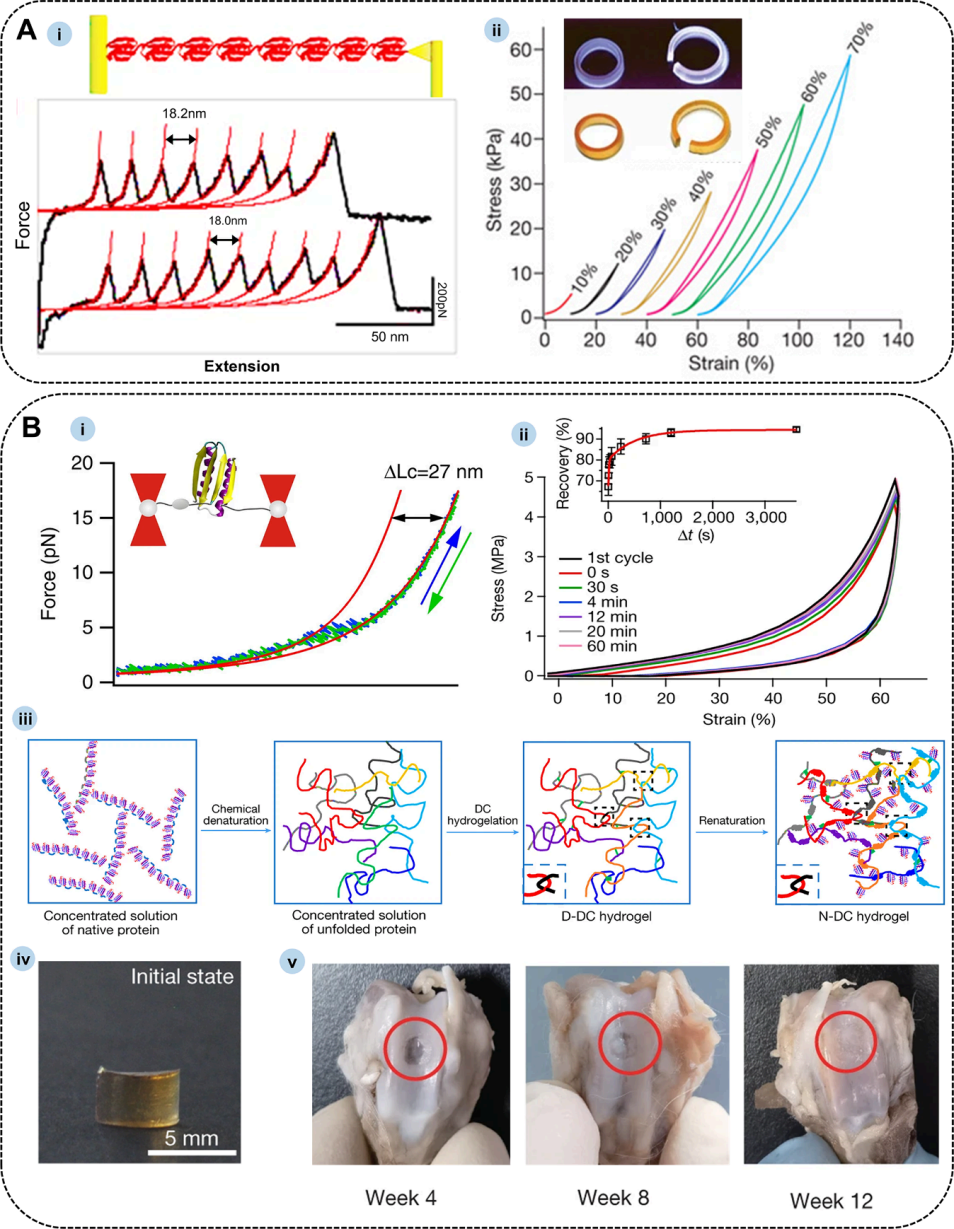
(A) (i) Typical force–extension curves of (GB1)_8_ polyproteins. Reproduced with permission from ref 99. Copyright 2007 Springer Nature Limited. (ii) Representative stress–strain curves of GB1-resilin-based biomaterials. Reproduced with permission from ref 73. Copyright 2010 Macmillan Publishers Limited. (B) (i) Representative force–extension curves of a single-FL domain. Reproduced with permission from ref 100. Copyright 2013 Springer Nature Limited. (ii) Consecutive compression–unloading cycles. (iii) Schematic illustration of the preparation protocol of the polyprotein biomaterial (the inset dashed square represents entangled chains). (iv) Photographs of (FL)_8_ elatomers. (v) The repair efficacy of osteochondral defects in a rabbit model at 4, 8, and 12 weeks post-implantation. Reproduced with permission from ref 101. Copyright 2023 Springer Nature Limited.

Building on this, Li et al.^73^ developed GB1-resilin-based biomaterials, which exhibit elastic properties and can be stretched up to 135% strain without breaking. The Young’s modulus of these materials ranges from 50 to 70 kPa (Figure 7A-ii), which is similar to the Young’s modulus measured for myofibrils/myocytes and falls within the physiological range of sarcomere length (60–100 kPa). As the sarcomere length increases, the hysteresis between stretching and relaxation also increases, mimicking the behavior of muscles as they act as shock absorbers at higher strains by efficiently dissipating energy. The mechanical behavior of GB1-resilin-based biomaterials differs significantly from that of traditional resilin-based biomaterials, emphasizing the critical role of the folded GB1 domain in determining the mechanical properties of the resulting materials. Upon stretching, the force-induced breakage of non-covalent bonds causes the unfolding of the GB1 β-sheet domain, leading to energy dissipation. Consequently, GB1 polyproteins show great promise as candidates for the development of artificial elastomeric proteins.^98^ Furthermore, their mechanical properties can be fine-tuned through protein engineering techniques, enabling customization to meet the specific demands of various applications.

#### 3.3.2 Elastomeric Materials Based on FL

Protein-based elastomeric materials often suffer from limited stretchability, which restricts their biological applications. This limitation is primarily due to the fact that unfolding typical elastomeric proteins requires forces in the range of hundreds of piconewtons (pN), with only a small fraction of the protein undergoing unfolding within the material. To overcome this challenge, Li et al.^100^ introduced a mechanically unstable elastomeric protein, FL. The FL protein, designed *de novo*, adopts a ferredoxin-like folding structure consisting of 82 amino acid residues arranged in a βαββαβ secondary structure along its backbone.^102^ The mechanical unfolding of FL occurs at approximately 5 pN, accompanied by a contour length increment (ΔLc) of around 27 nm, as shown in Figure 7B-i. This exceptionally low mechanical stability is a direct consequence of the protein’s structural topology. In the three-dimensional structure of FL, the two terminal load-bearing β-strands are positioned adjacently in an antiparallel arrangement. Upon mechanical stretching, these β-strands unfold in an unzipping fashion, causing the sequential disruption of the backbone hydrogen bonds that hold the strands together. This unzipping mechanism is characteristic of mechanically unstable proteins. Li et al.^103^ cross-linked FL protein in a denaturing solution, introducing entanglements during renaturation (Figure 7B-ii to Figure 7B-v). This strategy resulted in a protein-based elastomeric material with high stiffness and toughness. The biomaterial exhibited notable energy dissipation, rapid recovery, and mechanical properties suitable for potential use as cartilage replacement materials.

### 3.4 Elastomeric Material Based on SpyCatcher/SpyTag Topology

A promising approach in biomaterial design is the SpyCatcher/SpyTag system, which utilizes the spontaneous formation of an isopeptide bond between the SpyTag peptide (13 amino acids) and the SpyCatcher protein fragment (138 amino acids). This reaction occurs between Asp-117 of SpyTag and Lys-31 of SpyCatcher, reconstructing the immunoglobulin collagen adhesion domain CnaB2.^104^ Notably, the SpyCatcher-SpyTag reaction is highly efficient under mild physiological conditions (4–37 °C) without the need for additional chemical reagents or catalysts, making it particularly advantageous for biomaterial applications, as shown in Figure 8A.

**Figure 8:**
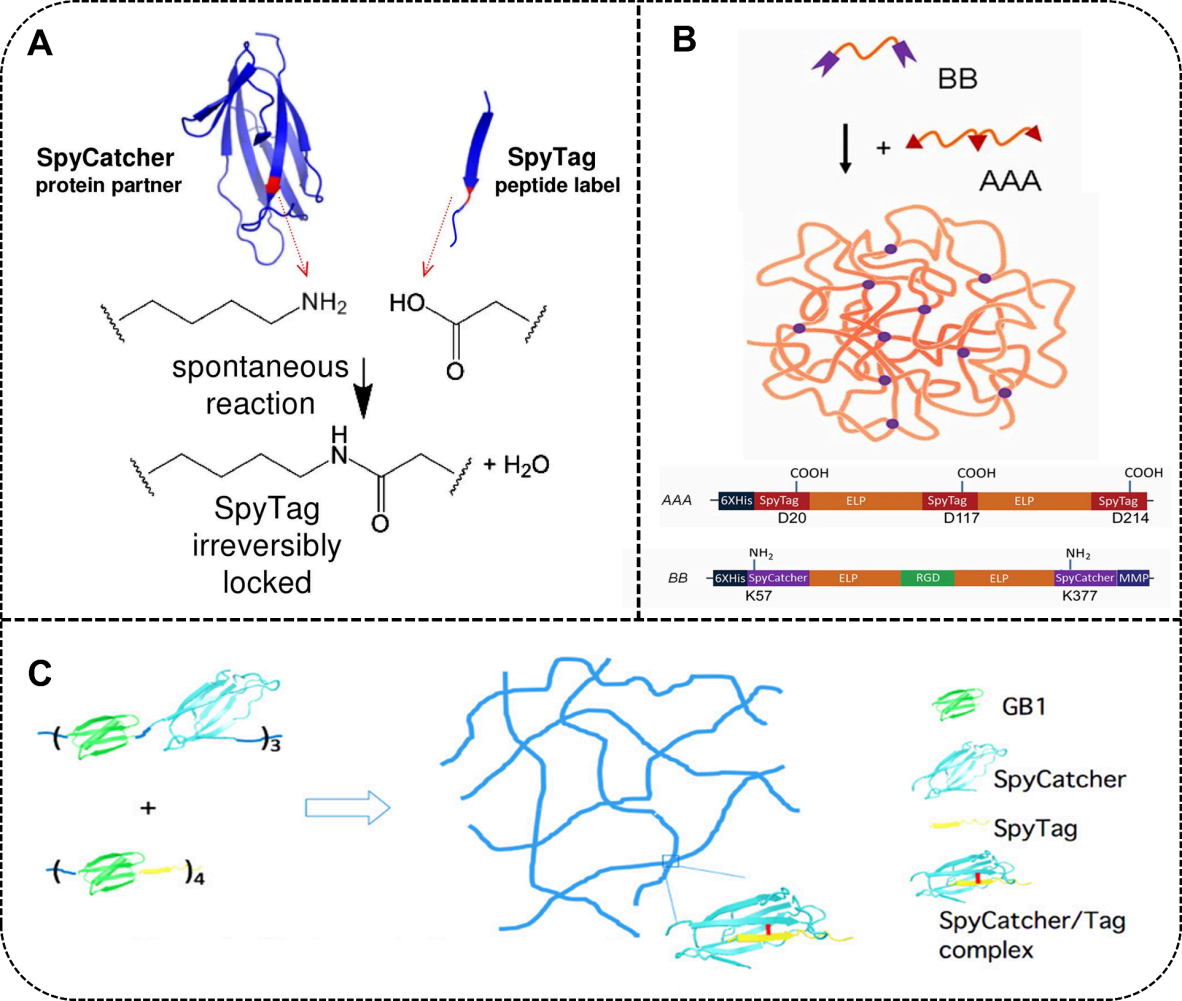
(A) Schematic diagram of SpyCatcher/SpyTag chemistry-mediated cross-linking. Reproduced from ref 104 with permission from the National Academy of Sciences. (B) SpyCatcher/ELP (BB) and SpyTag/ELP (AAA) tandem proteins to form covalently cross-linked, protein-based elastic materials. Reproduced from ref 105 with permission from the National Academy of Sciences. (C) (GSc)_3_ and (GSt)_4_ polyproteins readily form elastic materials. Reproduced from ref 106. Copyright 2016 American Chemical Society.

The system has been applied to design unconventional, nonlinear elastin-like protein (ELP) materials. For example, Tirrell et al.^105^ used multifunctional SpyCatcher/ELP (BB) and SpyTag/ELP (AAA) tandem proteins to form covalently cross-linked, protein-based elastic materials under physiological conditions (Figure 8B). These Spy networks are fully genetically encoded, enabling the seamless incorporation of multiple functional protein motifs without the need for chemical modifications. For instance, networks incorporating the fluorescent protein mCherry and the cell adhesion peptide RGD were successfully used in 3T3 fibroblast encapsulation and 3D stem cell culture applications.

In addition to elastin-like proteins, structurally folded globular domains, such as the GB1 and FnIII domains, have been explored as building blocks for Spy networks. Li et al.^107^ incorporated these folded domains into elastic materials, which exhibited enhanced mechanical properties (Figure 8C). These materials were successfully used for the 3D encapsulation and culture of human lung fibroblasts, as well as for drug loading and release applications. The SpyCatcher-SpyTag system offers an efficient, biocompatible, and highly modular platform for designing protein-based materials. However, unlike split intein-catalyzed polymerization, the SpyCatcher/SpyTag reaction leaves a significant residual sequence (up to 100 amino acids). These non-native residues may introduce steric hindrance during protein assembly and reduce chain flexibility due to the inherent rigidity of the SpyCatcher domain. Therefore, when employing this approach, it is critical to evaluate how these structural elements affect local conformations and bulk material properties.

## 4 Conclusion and Future Perspectives

Synthetic polymers derived from fossil fuels pose significant environmental risks and hinder biomedical applications due to their poor degradability, low recyclability, and poor biocompatibility, necessitating the urgent development of sustainable alternatives. Natural protein biopolymers synthesized by living cells provide valuable inspiration.^108,109^ In particular, the natural elastomeric proteins produced through biological evolution exhibit exceptional mechanical properties (such as elasticity, resilience, and fatigue resistance) as well as excellent biocompatibility, making them highly promising for biomedical applications. Understanding the sequence-structure-property relationships of natural elastomeric proteins not only aids in unraveling the origins of elasticity but also serves as the theoretical foundation for designing and developing elastomeric materials. With advancements in synthetic biology, researchers are now capable of engineering artificial elastomeric proteins with diverse structures and thus properties through sequence manipulation.^110^ In general, random coil structures offer great flexibility and fatigue resistance, while the α-helical and β-sheet structures of proteins are conductive to high structural stability, mechanical strength, and the capacity to resist or recover from large deformation. Protein polymerization and cross-linking constitute critical processes for engineering mechanically resilient and tough materials. Physical cross-linking networks are predominantly formed by transient connections arising from chain entanglements or non-covalent interactions, such as hydrogen bonds, ionic interactions, and hydrophobic forces. In contrast, chemical cross-linking is achieved through the formation of covalent bonds involving amino acids with reactive side chains, like tyrosine, lysine, and cysteine. Despite considerable advances in elastomeric protein engineering, fundamental challenges remain in the predictive design of high-performance elastomeric materials and their translation into viable industrial and biomedical applications.

The rational design of high-performance protein elastomers is hindered by the intricate sequence-structure-function relationship and challenges in translating molecular properties to macroscopic behavior. Computational strategies now address these limitations through complementary approaches. First, machine learning tools (e.g., RFdiffusion, ProteinMPNN, and RoseTTAFold)^111,112^ leverage large-scale datasets to generate programmable elastomeric sequences using advanced architectures like protein language models and graph neural networks, accelerating the discovery of novel proteins beyond the limitations of natural templates. Second, molecular dynamics and coarse-grained simulations reveal mechanical mechanisms underlying molecular cross-linking, conformational transitions, and sequence motifs. Furthermore, simulation-derived features (e.g., residue fluctuations, stiffness networks, structural transitions) enhance machine learning models, enabling synergistic frameworks for high-throughput variant screening and predictive design.^113^

Achieving an optimal balance between elasticity and mechanical strength in protein elastomers represents a fundamental challenge, as these two properties are intrinsically antagonistic. Elasticity arises from molecular mobility and conformational flexibility, whereas mechanical strength typically requires rigid, well-organized structures. Current design strategies to reconcile this balance include modular engineering that integrates flexible and rigid domains, as well as tuning protein chain length and precisely controlling cross-linking sites. Nanocomposite reinforcement utilizing nanofillers such as carbon nanotubes and nanocellulose provides a promising approach to enhance mechanical strength while preserving material extensibility. However, homogeneous nanofiller dispersion is essential to prevent stress concentration and subsequent embrittlement.^114^ Moreover, multiscale hierarchical architectures, spanning molecular to macroscopic levels, have been shown to synergistically improve both toughness and elasticity. Collectively, these approaches establish a rational framework for tailoring protein elastomers to meet application-specific mechanical demands.

Moreover, the lack of standardized mechanical testing protocols introduces significant variability in reported results due to inconsistencies in sample preparation, hydration state, strain rate, and methodology (e.g., tensile testing vs. DMA). Tensile tests at constant strain rate, while suitable for large-strain quasi-static deformation, fail to capture time-dependent viscoelastic effects. Alternatively, DMA provides storage/loss moduli and estimates resilience via the damping coefficient (tan δ) of the material. However, DMA is generally performed under small-strain oscillatory conditions. Resilience can be accurately calculated from tan δ when tan δ < 0.1, but may be overestimated at higher values. Drop-ball impact tests, though effective for bulk elastomers under high-strain-rate conditions, are impractical for protein-based materials due to limited sample volumes and geometric constraints. Therefore, establishing universally accepted guidelines would substantially enhance data reproducibility and enable meaningful comparisons across studies, thereby accelerating both fundamental research and technological translation.

The translational application of protein-based biomaterials faces key practical challenges including cost-effectiveness and regulatory compliance. Although synthetic biology advances, such as genome-wide strain engineering, pathway optimization, and chaperone co-expression, have boosted recombinant protein yields, large-scale manufacturing remains costly. A promising strategy to enhance cost efficiency involves engineering microbial hosts to utilize low-value substrates (e.g., methanol, acetate, or agricultural waste), which could improve affordability and sustainability. For biomedical applications, comprehensive evaluation of biocompatibility, immunogenicity, purity, and degradation kinetics is essential. Protein products containing non-native residues, aberrant post-translational modifications, or host cell-derived contaminants may elicit adverse immune responses or pose safety risks, thereby complicating regulatory approval. Consequently, comprehensive regulatory compliance necessitates systematic in vitro and in vivo biosafety testing combined with strict adherence to Good Manufacturing Practice (GMP) standards to ensure product quality and safety.

In conclusion, protein-based elastomers emerge as a promising class of materials for sustainable biomedical engineering, combining exceptional mechanical performance with inherent biocompatibility and ecological benefits. While significant progress has been made in understanding molecular design principles, optimizing mechanical properties, developing computational models, and regulating biosynthetic pathways, key challenges remain in scalable production, standardized testing, and clinical translation. Future progress in machine learning-assisted protein engineering, synthetic biology platforms, advanced characterization techniques and harmonized regulatory standards present transformative potential to overcome current limitations, enabling more efficient production and broader commercial adoption.
